# Supplementary material for: Weakening of resistance force by cell–ECM interactions regulate cell migration directionality and pattern formation
Source: Commun Biol. 2021 Jun 28;4:808. doi: 10.1038/s42003-021-02350-4 (PMC8239002; doi:10.1038/s42003-021-02350-4)
Supplement: Supplementary file 2 — Supplementary Information [file 42003_2021_2350_MOESM2_ESM.pdf]

Supplementary Information

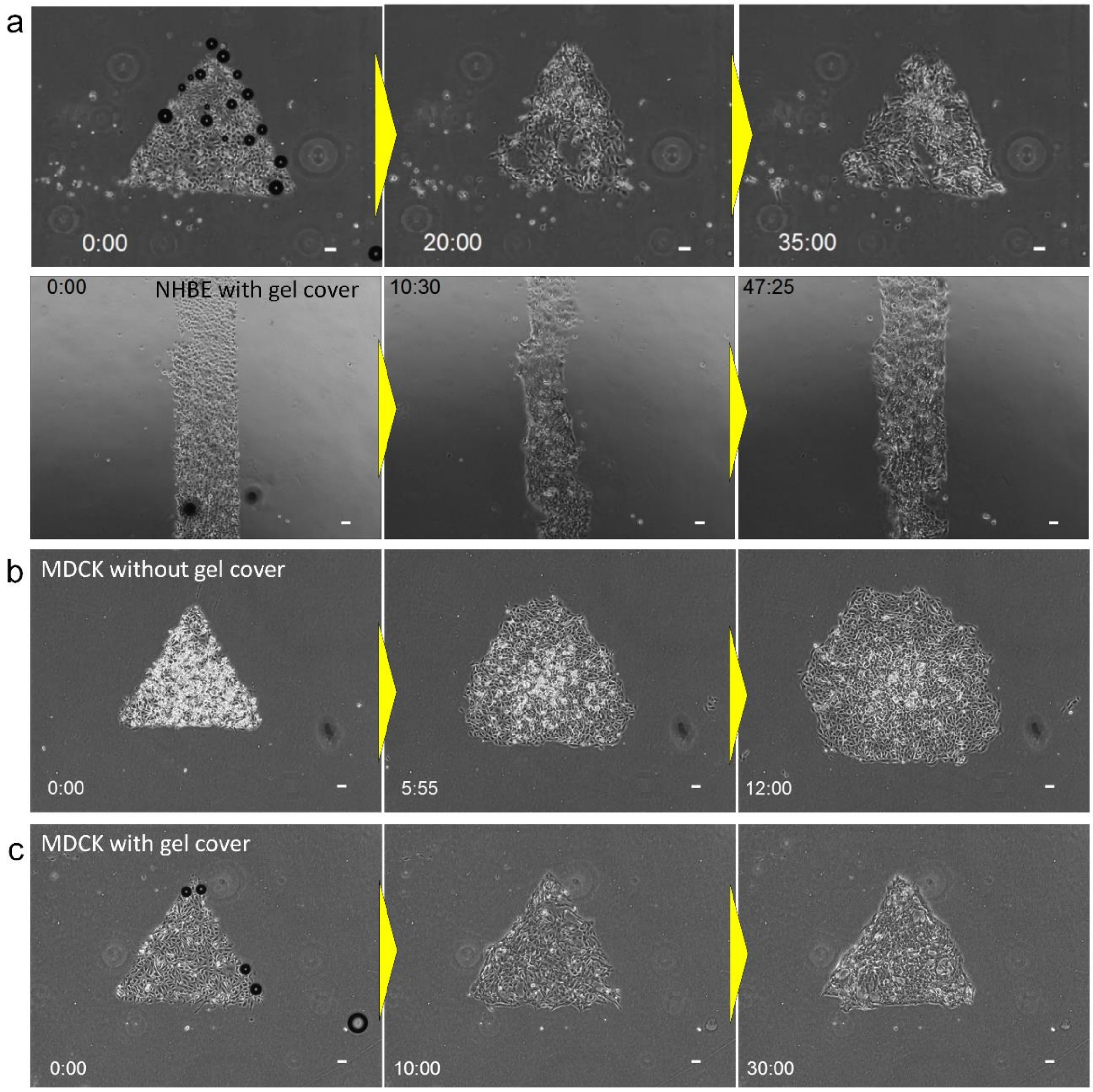

Supplementary Fig. 1: Phase contrast time-lapse images of NHBE and MDCK cells. **a,** Phase contrast time-lapse images of NHBE cells patterned in a triangle and long rectangle. The initial geometry of the collective cells collapsed after 10 -20 hours, but returned to the original shape after 35 - 40 hours. The black dots are air bubbles generated during gelation. Regardless of cell pattern geometry or size, the cells could recover the initial geometry after a certain period of time. The full time-lapse movies are shown in Supplementary videos 2 and 3. **b, c** Phase contrast time-lapse images of MDCK cells patterned in a triangular shape with and without Matrigel, respectively. Without the gel, the cells readily exited the initial triangular area and expanded their territory. On the other hand, in the presence of a gel, the cells could not move outside of the initial triangular shape. The complete time-lapse movies are shown in Supplementary videos 4. Scale bar: 100 μm.

Supplementary Information

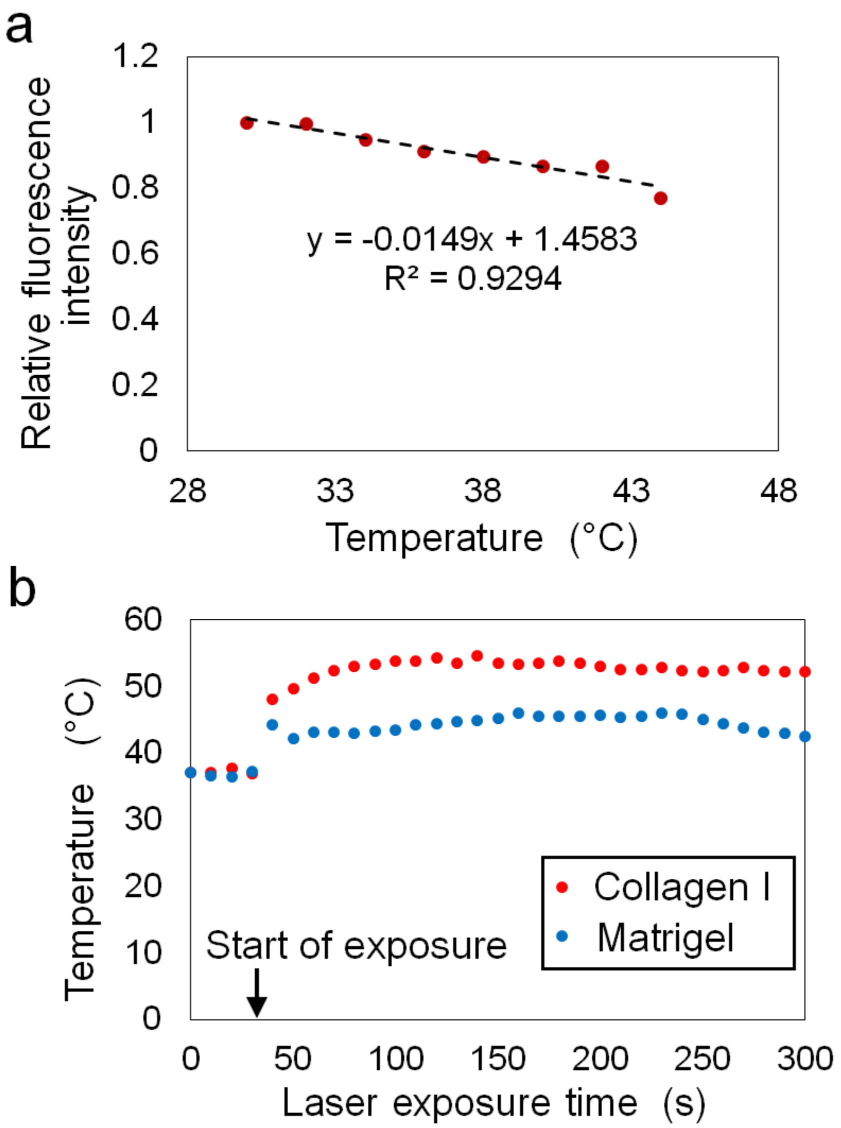

Supplementary Fig. 2: Measurement of temperature changes using Rhodamine B as an optical thermal sensor. **a,** Calibration data of Rhodamine B fluorescence intensity over temperature change. Linear correlation between water temperature and fluorescence intensity was confirmed. **b,** Measurement of temperature in Matrigel and collagen I at laser point. The laser was turned on 30s after the measurement had started. An increase of approximately 9 and 17 °C was detected in Matrigel and collage, respectively, at the point of laser exposure.

Supplementary Information

Calibration of optical tweezers to calculate resistance force in the ECM

The microbeads were subjected to a traction force when the focused laser was sufficiently close to the optical tweezers. The amount of force increases linearly with increasing distance from the centre of the laser to the microbeads, which is similar to the force of a spring and can thus be expressed as follows:

$$F_{trap} = kx \qquad (1)$$

where k is the spring coefficient of the system, and x is the distance from the laser to the trapped microbeads. Therefore, once the system-dependent constant k is determined, the applied trap force in the ECM or resistance force from the ECM can be calculated from the distance x. When the microbeads are manipulated in a liquid environment, a drag resistance force is applied and can be calculated by Stokes's law as follows:

$$F_d = \frac{6\pi\eta R v}{\left\{1-\frac{9}{16}\left(\frac{R}{h}\right)+\frac{1}{8}\left(\frac{R}{h}\right)^3-\frac{45}{256}\left(\frac{R}{h}\right)^4-\frac{1}{16}\left(\frac{R}{h}\right)^5\right\}} \qquad (2)$$

where η, R, v, and h represent the dynamic viscosity, radius of the sphere, velocity, and bead height from the substrate, respectively. During the manipulation of microbeads by the optical tweezers, the trap force (F_{trap}) and drag force (F_d) were balanced as shown in Supplementary Fig. 3a. Subsequently, the spring coefficient k was calculated as follows:

$$k = \frac{6\pi\eta R v}{x\left\{1-\frac{9}{16}\left(\frac{R}{h}\right)+\frac{1}{8}\left(\frac{R}{h}\right)^3-\frac{45}{256}\left(\frac{R}{h}\right)^4-\frac{1}{16}\left(\frac{R}{h}\right)^5\right\}} \qquad (3)$$

To calculate the k value in the current optical tweezer system, calibration data were obtained in a water environment as shown in Supplemental Fig. 3b, and the k value was obtained from the slope in Supplemental Fig. 3c, which was 67.63 pN/μm.

Supplementary Information

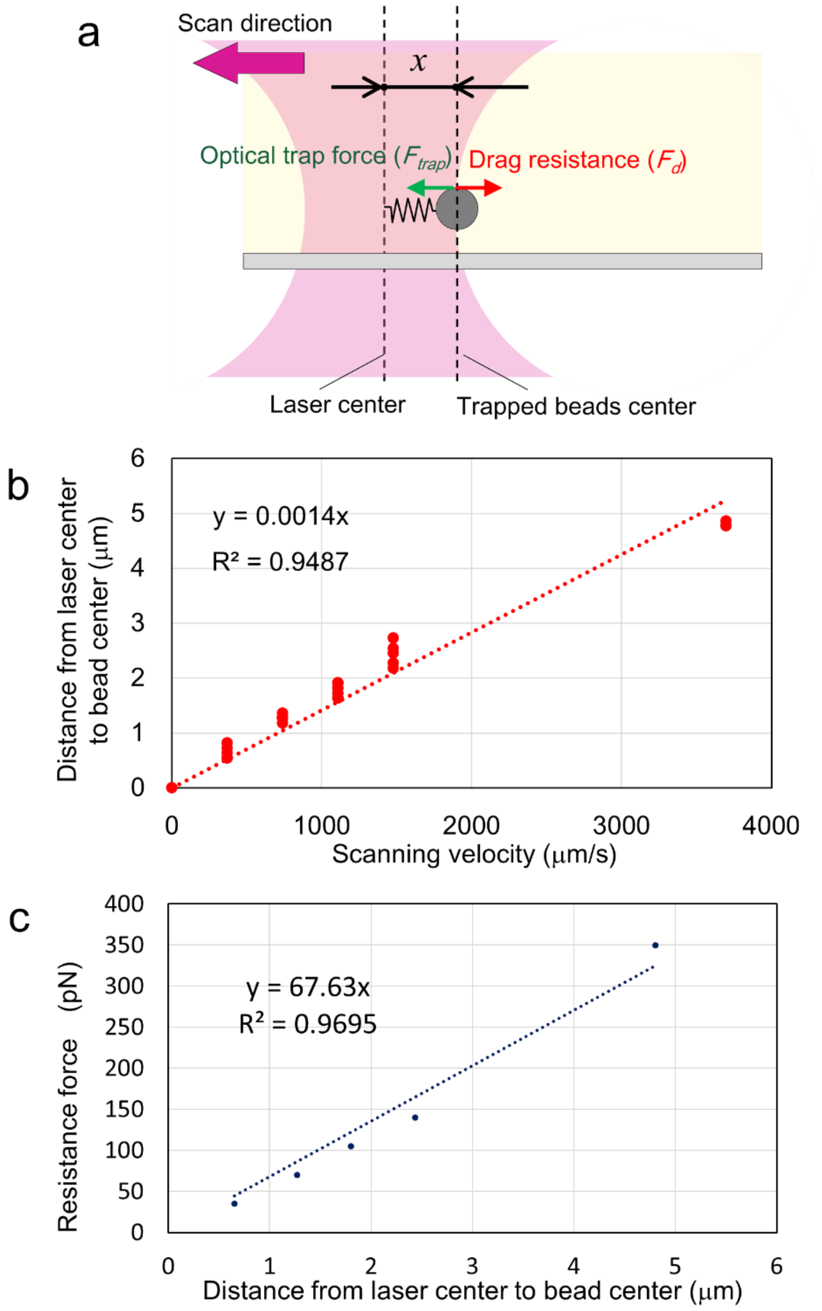

Supplementary Fig. 3: Calibration data to calculate the resistance force in the ECM by optical tweezers. **a,** Schematic representation to illustrate the applied force and variables during microbead manipulation by optical tweezers. **b,** Calibration data of the correlation between the scanning velocity and laser-to-bead distance when 10 µm microbeads were manipulated by optical tweezers in water. **c,** Correlation between resistance force, which was calculated by Stokes's law, and the measured distance between the laser centre and bead centre. The optical spring coefficient *k* was found to be 67.63 pN/µm

Supplementary Information

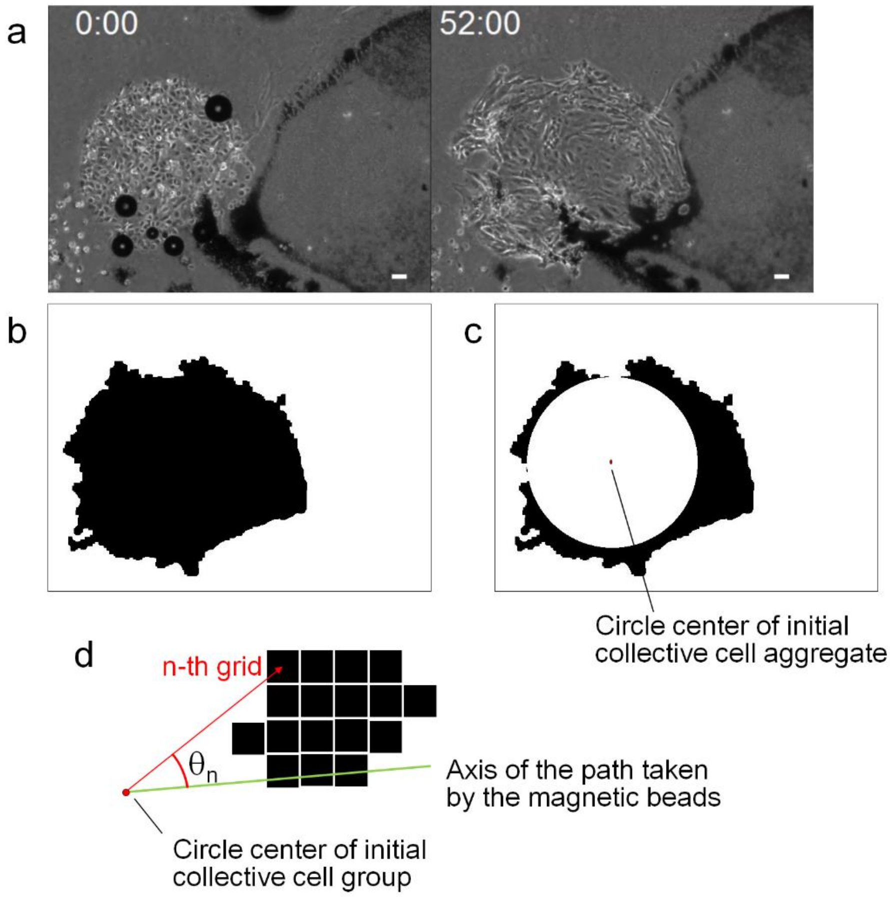

Supplemental Fig. 4: Procedure for quantification of directionality in collective cell migration towards the area degraded by magnetic bead movement. **a,** Phase contrast time-lapse images of cells patterned in a circular shape at the start of culture and after 52 hours of culture. **b,** Generated binary image. **c,** Binary image with subtracted circular shape of initial cell culture position. **d,** Calculation of angles for all grids from the axis of the path taken by the magnetic beads.

Supplementary Information

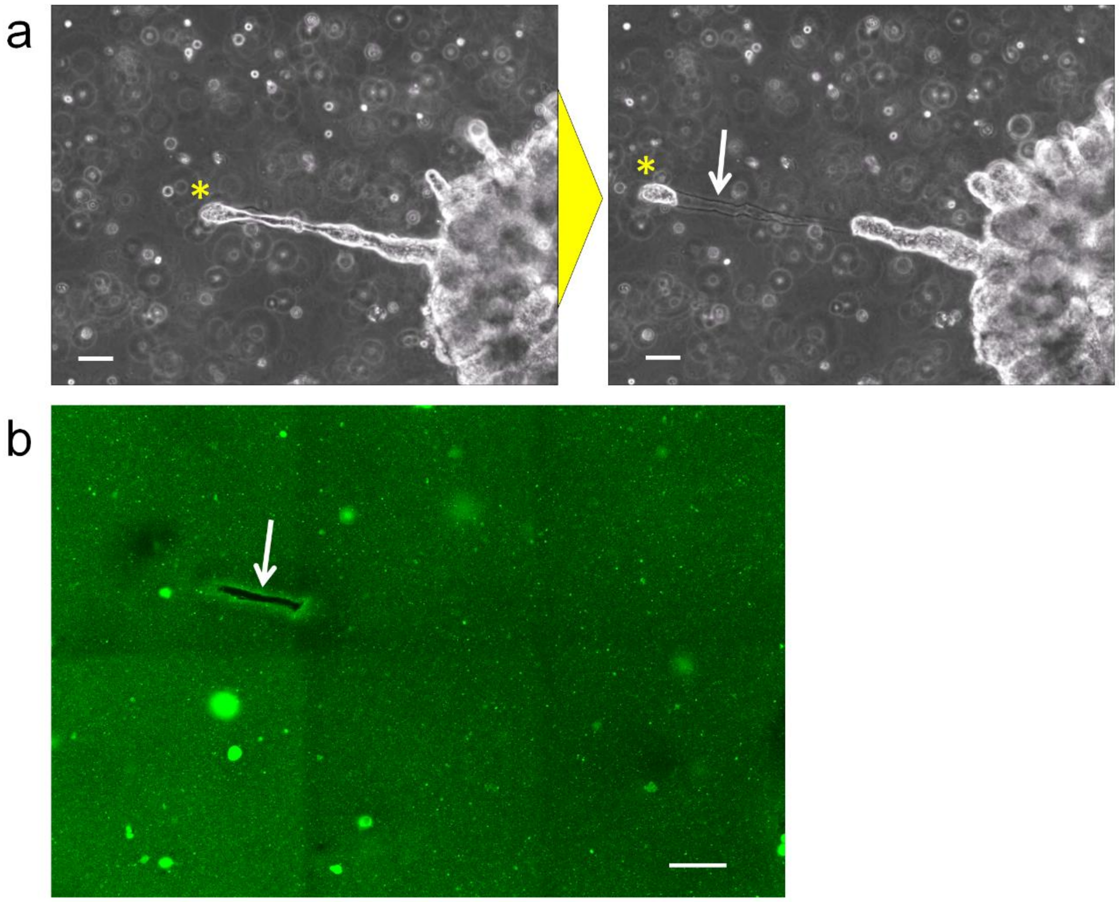

Supplemental Fig. 5: Time-lapse and z-stack imaging of 3D NHBE branching with DQ collagen in Matrigel. **a,** time-lapse imaging of NHBE branching in Matrigel supplemented with DQ collagen IV. Leader cells were emerged and branches were generated rapidly from NHBE cell clot. Then the leader cells were shot out from the branch while the cell path was remained in the Matrigel. **b,** xy spatial tiling image at the specific z height, taken by Z-stack imaging of the path of leader cells with DQ collagen. The tunnel was clearly observed at the path of the leader cells. The whole time-lapse and z-stack imaging is available in the supplementary video 17. Scale bar: 100 μm.

Supplementary Information

Theoretical model of cellular behaviour in 2D.

A particle model has been developed to simulate the cellular behaviour in the ECM. The model includes the phenomena of cell polarity and cell-cell adhesion as described by Akiyama et al.[1], reaction-diffusion chemotaxis as described in previous work [2], as well as the addition of resistance force from the surrounding ECM. In NHBE cell migration, the effects of chemotaxis affected is more prominent when there is a large number of surrounding cells and when the secretion ratio of activator and inhibitor is quite small. Therefore, the model in this paper was designed based on a large population of cells. The polarity takes into account continuous cell movement, and cell-cell adhesion force was contributed by the neighbouring cells, while chemotaxis was determined by the distribution of a large number of cells. The cell-ECM adhesion force could be another resistance force affecting cell migration, but as the Matrigel is covered on the top of the cell layer, the applied resistance force by cell-ECM adhesion is assumed to be consistent in the presented experimental assay. In that sense, the effects of cell-ECM adhesion on cell directionality should account for less than the other phenomena considered in the model. Therefore, in order to simplify the calculations, we opt to omit the effects of cell-ECM in this paper. Thus, the total force applied on *i-th* cell is expressed as the sum of the four terms.

$$\boldsymbol{F_i} = \alpha \boldsymbol{F^i_{polarity}} + \beta \boldsymbol{F^i_{adhesion}} + \gamma \boldsymbol{F^i_{chem}} + \delta \boldsymbol{F^i_{ECM}} \qquad (4)$$

The individual terms in equation (4) are explained in the following sections.

1) Cell polarity

Cells possess a front-back polarity, and they can migrate based on the direction of this polarity without any external force being applied. $\boldsymbol{F_{polarity}}$ expresses this inherent driving force determined by the following equations.

$$\boldsymbol{F^i_{polarity}} = \begin{pmatrix} cos\ \theta_i \\ sin\ \theta_i \end{pmatrix} \qquad (5)$$

The polarity direction θ_i is affected by the polarities of neighbouring cells and their direction of migration. The polarities of cells are gradually altered to correspond with that of their neighbouring cells, as well as their direction of migration (Supplementary Fig. 6). Thus, the change in cell polarity over time is expressed as

$$\boldsymbol{\dot{\theta}_i} = \xi \boldsymbol{G^i_{share}} + \eta \boldsymbol{G^i_{motion}} \qquad (6)$$

where $\boldsymbol{G^i_{share}} = \sum_{i \neq j} g(|\boldsymbol{r_{i,j}}|)\, sin(\theta_j - \theta_i)$

$$g(|\boldsymbol{r_{i,j}}|) = \begin{cases} 1 & (|\boldsymbol{r_{i,j}}| < R_T) \\ 0 & (otherwise) \end{cases}$$

$$\boldsymbol{G^i_{motion}} = \sin(\mathrm{Arg}(\boldsymbol{F_i} - \theta_i))$$

$\boldsymbol{r_{i,j}}$ is the vector from the *i-th* cell to the *j-th* cell, and R_T is the maximum cell diameter at which the cell is subject to adhesion forces.

Supplementary Information

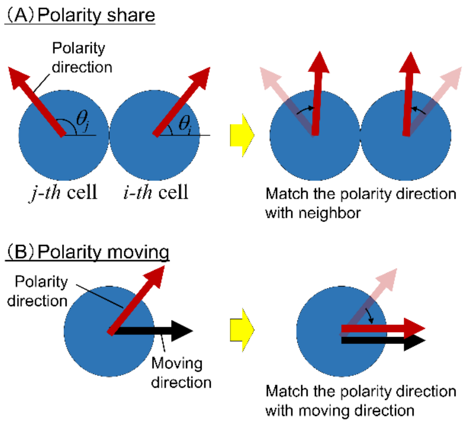

Supplementary Fig. 6 Schematic illustration of changes in cell polarity. (A) Polarity sharing. The direction of cell polarity direction shifts to correspond with that of its neighbour cells. (B) Moving polarity. The direction of cell polarity shifts to correspond with the direction of movement.

2) Cell adhesion

Cell adhesion force is another major contributor that determines cellular behaviour. Cells are subject to tensile or compression forces from their neighbour cells depending on their physical distance. Thus, the total adhesion force applied on the *i-th* cells is

$$\boldsymbol{F}^{i}_{\boldsymbol{adhesion}} = \sum \frac{\boldsymbol{r}_{i,j}}{|\boldsymbol{r}_{i,j}|} \begin{cases} 2p(|\boldsymbol{r}_{i,j}| - R_A), & |\boldsymbol{r}_{i,j}| \in (0, R_A) \\ 2q(R_T - |\boldsymbol{r}_{i,j}|), & |\boldsymbol{r}_{i,j}| \in [R_A, R_T) \\ 0, & |\boldsymbol{r}_{i,j}| \in [R_A, R_T) \end{cases} \quad (7)$$

where R_A is the diameter of cell size, and the values p and q are constants that determine the strength of tensile or compression forces. Cells exert a repulsive force when their distance to another cell is less than their original size, and experience a pulling force when the distance is more than the original size but less than the maximum adhesion length (Supplementary Fig. 7).

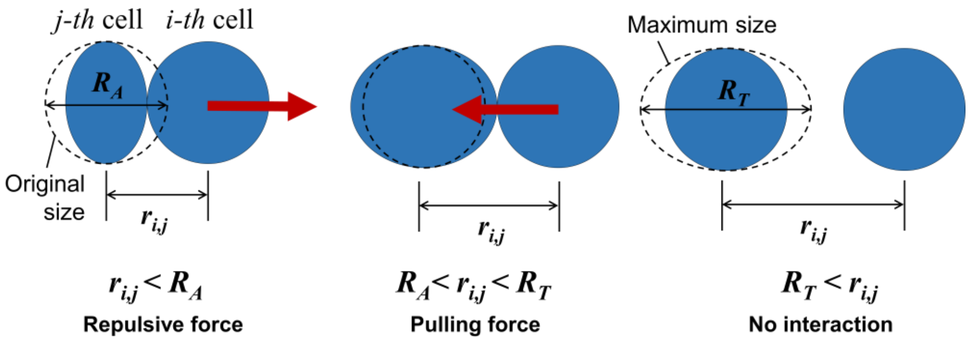

Supplementary Fig. 7 Schematic illustration of adhesion force. Physical cell-cell contact generates either a repulsive or pulling force, depending on the distance between neighbouring cells.

Supplementary Information

3) Chemotaxis

Chemotaxis is also a major driving force for cells to migrate. We consider that cells are trying to migrate towards regions with a higher concentration of activator, and the magnitude of the driving force generated is proportional to the activator gradient as shown in equation (8) (Supplementary Fig. 8). In this model, the reaction-diffusion system based on H. Meinhardt's model (H. Meinhardt, Differentiation, 6, 1976) was employed to calculate the distribution of morphogen in the culture area, since it had good agreement with the behaviour of NHBE cells as described in our previous work[32]. Four variables were taken into account: the concentrations of an activator (A), an inhibitor (H), a substrate chemical (S), and a biological marker (Y). Activator A has an auto-catalyst function and also stimulates the production of inhibitor H, at rate c. Substrate S is required for the production of morphogens [cA^2S in equations (9) and (10)], and inhibitor H also regulates activator production [cA^2S/H in equation (9)]. The production of morphogens A and H are downregulated at rates μ and ν, respectively, and are also secreted by cells at rates ρ_A and ρ_H [$\rho_A Y$ and $\rho_H Y$ in equations (9) and (10)], respectively. S is consumed by cells at a rate of ε [εSY in equation (11)]. Y supplies 0 or 1 values depending on the presence of cells. If cell i is present at a position $X_i = (x_i, y_i)$ and there are no other cells in the culture area, then $Y = 1$ will be marked at this position while all other regions are marked as 0. Morphogens A, H, and S diffuse to the surrounding area at rates D_A, D_H and D_S, respectively. The parameters ρ_A, ρ_H and ε are determined based on the genetic program of the cells. Other parameters are determined by environmental factors such as temperature, pressure and ECM stiffness. For example, if the stiffness of the ECM is increased, the mesh size inside the ECM becomes smaller, and D_A, D_H and D_S become lower.

$$\boldsymbol{F^i_{chem}} = \boldsymbol{\nabla A} \qquad (8)$$

$$\frac{\partial \boldsymbol{A}}{\partial t} = \frac{c\boldsymbol{A^2S}}{\boldsymbol{H}} - \mu\boldsymbol{A} + D_A\boldsymbol{\nabla}^2\boldsymbol{A} + \rho_A\boldsymbol{Y} \qquad (9)$$

$$\frac{\partial \boldsymbol{H}}{\partial t} = c\boldsymbol{A^2S} - \nu\boldsymbol{H} + D_H\boldsymbol{\nabla^2 H} + \rho_H\boldsymbol{Y} \qquad (10)$$

$$\frac{\partial \boldsymbol{S}}{\partial t} = -\varepsilon\boldsymbol{YS} + D_S\boldsymbol{\nabla^2 S} \qquad (11)$$

$$\mathbf{Y} = \sum_{i=1}^{n}\int_\Omega \delta|\boldsymbol{X} - \boldsymbol{X_i}|d\boldsymbol{X} \qquad (12)$$

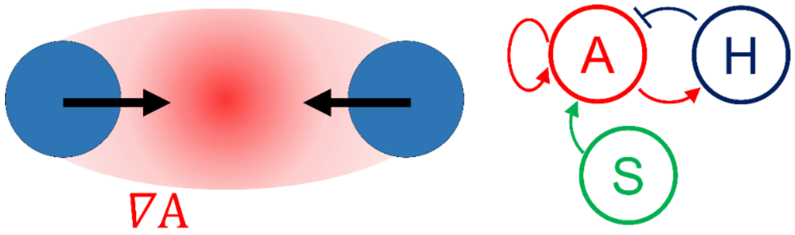

Supplementary Fig. 8 Schematic illustration of morphogen interactions and chemotaxis. Cells are driven to migrate towards areas with higher concentration of activator *A* by chemotactic forces.

Supplementary Information

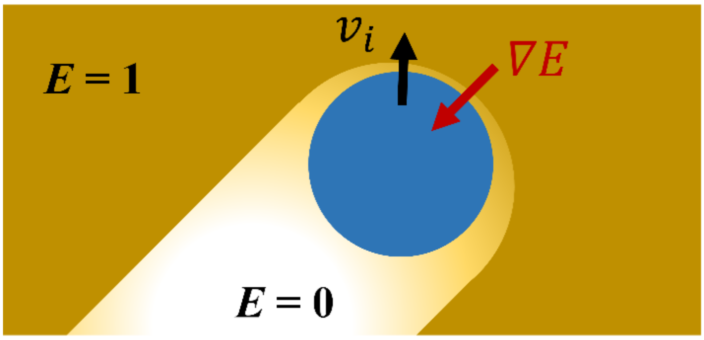

Supplementary Fig. 9 Schematic image of resistance force from the surrounding environment. The resistance force field (*E*) is dynamically degraded by the presence of cells.

4) ECM resistance force

The resistance force from the ECM is also taken into account in this model. Based on the experimental results, cellular movement dynamically degrades the field of ECM resistance force, and once cells start to explore new sites, the resistance force will be further weakened so that cells can expand their territory. We consider that the amount of resistance force should be proportional to the velocity of the cells and the gradient of this resistance force field (Supplementary Fig. 9). Thus, the resistance force applied on the cell is expressed as

$$\boldsymbol{F}^{i}_{ECM} = -(\nabla \boldsymbol{E} \cdot \boldsymbol{v}_i)\nabla \boldsymbol{E} \qquad (13)$$

where $\boldsymbol{v}_i$ and $\boldsymbol{E}$ denote the velocity of *i-th* cell and the resistance force field, respectively. In order to update the resistance force field (E) based on cellular movement, we introduce the function (h) to denote the amount of degradation based on cellular location.

$$h(r) = \frac{\tanh(\mu(r_0 - r)) + 1}{2} \qquad (14)$$

h(r) gives a value in between 0 and 1, depending on the distance (r) from the centre of the cell . μ and r_0 denote the parameters determining the transition curvature between 0 and 1. Then, the updated resistance force field ($\boldsymbol{E}$), at time t, can be expressed as follows:

$$\boldsymbol{E}_t(x, y) = \begin{cases} 0 & (\boldsymbol{H}_t > 1) \\ 1 - H_t & (\boldsymbol{H}_t \leq 1) \end{cases} \qquad (15)$$

where $\boldsymbol{H}_t(x, y) = \sum_i h\left(\sqrt{\left(r_x^i - x\right)^2 + (r_y^i - y)^2}\right) + H_{t-1}(x, y)$

r_x^i and r_y^i denotes the x and y components of centre position of the *i-th* cell.

Based on the total force ($\boldsymbol{F}_i$) applied on *i-th* cell in equation (4), the velocity of the cell is considered as follows:

$$\dot{\boldsymbol{r}}_i = f(|\boldsymbol{F}_i|)\boldsymbol{F}_i \qquad (16)$$

$$\boldsymbol{f}(|\boldsymbol{F}_i|) = \frac{V_{max}}{|\boldsymbol{F}_i|}\tanh\left(\frac{\rho|\boldsymbol{F}_i|}{V_{max}}\right) \qquad (17)$$

$\boldsymbol{f}(|\boldsymbol{F}_i|)$ supplies a non-linear function to saturate the value at v_{max} with increasing $|\boldsymbol{F}_i|$, while it

is approximately a linear function when $|\boldsymbol{F_i}|$ is low, and ρ is a parameter to determine the non-linearity.

In the computational simulation, the calculation area was taken as 3384 × 3384 μm, divided by 200×200 grids. Then, the following experimental constants were used:

$\alpha = 6$, $\beta = 2.5$, $\gamma = 2$, $\delta = 0.45$, $\xi = 1$, $\eta = 2$, $p = 1$, $q = 0.4$, $R_A = 45$, $R_T = 50$, $\rho = 2$, $v_{max} = 2.5$.

$c = 0.016$, $\mu = 0.1315$, $\rho_A = 0.05$, $\rho_H = 0.088$, $\eta = 0.04$, $\varepsilon = 0.52$, $D_A = 8.6$, $D_H = 2556$, $D_S = 37.2$.

Cell proliferation was also taken into account in the simulation, and the number of cells were increased exponentially as expressed as $n = n_0 \times 2^{kt}$, where n and n_0 indicate the total and initial cell number, respectively, and k is determined by the proliferation rate of the cell.
