# Supplementary material for: Weakening of resistance force by cell–ECM interactions regulate cell migration directionality and pattern formation
Source: Commun Biol. 2021 Jun 28;4:808. doi: 10.1038/s42003-021-02350-4 (PMC8239002; doi:10.1038/s42003-021-02350-4)
Supplement: Supplementary file 3 — Description of Additional Supplementary Files [file 42003_2021_2350_MOESM3_ESM.pdf]

Description of Additional Supplementary Files

File Name: Supplementary Data 1
Description: Collected data to quantify the resistance force by optical tweezers.

File Name: Supplementary Data 2
Description: Collected data of cell movement area expansion rate with different Matrigel stiffness.

File Name: Supplementary Movie 1
Description: Cellular behavior at FN boundary.

File Name: Supplementary Movie 2
Description: Collective cell behavior at FN boundary.

File Name: Supplementary Movie 3
Description: Collective cell behavior at ex-boundary of PDMS membrane without FN pattern.

File Name: Supplementary Movie 4
Description: NHBE cell migration without Matrigel cover. (Circular pattern)

File Name: Supplementary Movie 5
Description: NHBE cell migration with Matrigel cover. (Circular pattern)

File Name: Supplementary Movie 6
Description: NHBE cell migration with Matrigel cover. (Various geometric patterns)

File Name: Supplementary Movie 7
Description: MDCK cell migration without and with Matrigel cover.
File Name: Supplementary Movie 8
Description: 3D imaging of Actin and nucleus for visualizing 3D cell organizations

File Name: Supplementary Movie 9
Description: Individual NHBE cellular behaviour without Matrigel cover.

File Name: Supplementary Movie 10
Description: Individual NHBE cellular behaviour with Matrigel cover.

File Name: Supplementary Movie 11
Description: Simulation result without resistance force from ECM.

File Name: Supplementary Movie 12
Description: Simulation result with resistance force from ECM.

File Name: Supplementary Movie 13
Description: Directing collective cell migration by artificial modification of ECM with magnetic microbeads.

File Name: Supplementary Movie 14
Description: Simulation result without artificial modification in ECM.

File Name: Supplementary Movie 15
Description: Simulation result with artificial modification in ECM.

File Name: Supplementary Movie 16
Description: 3D NHBE branching morphogenesis in the Matrigel localized 2 different stiffness.

File Name: Supplementary Movie 17
Description: Time-lapse and z-stack imaging of 3D NHBE branching with DQ collagen in Matrigel
