# Supplementary material for: Weakening of resistance force by cell–ECM interactions regulate cell migration directionality and pattern formation
Source: Commun Biol. 2021 Jun 28;4:808. doi: 10.1038/s42003-021-02350-4 (PMC8239002; doi:10.1038/s42003-021-02350-4)
Supplement: Supplementary file 23 — Reporting Summary [file 42003_2021_2350_MOESM23_ESM.pdf]

nature research

Corresponding author(s): Masaya Hagiwara

Last updated by author(s): May 17, 2021

Reporting Summary

Nature Research wishes to improve the reproducibility of the work that we publish. This form provides structure for consistency and transparency in reporting. For further information on Nature Research policies, see our Editorial Policies and the Editorial Policy Checklist.

Statistics

For all statistical analyses, confirm that the following items are present in the figure legend, table legend, main text, or Methods section.

n/a	Confirmed	
☐	☒	The exact sample size (n) for each experimental group/condition, given as a discrete number and unit of measurement
☐	☒	A statement on whether measurements were taken from distinct samples or whether the same sample was measured repeatedly
☐	☒	The statistical test(s) used AND whether they are one- or two-sided<br>*Only common tests should be described solely by name; describe more complex techniques in the Methods section.*
☒	☐	A description of all covariates tested
☐	☒	A description of any assumptions or corrections, such as tests of normality and adjustment for multiple comparisons
☐	☒	A full description of the statistical parameters including central tendency (e.g. means) or other basic estimates (e.g. regression coefficient) AND variation (e.g. standard deviation) or associated estimates of uncertainty (e.g. confidence intervals)
☐	☒	For null hypothesis testing, the test statistic (e.g. F, t, r) with confidence intervals, effect sizes, degrees of freedom and P value noted<br>*Give P values as exact values whenever suitable.*
☒	☐	For Bayesian analysis, information on the choice of priors and Markov chain Monte Carlo settings
☒	☐	For hierarchical and complex designs, identification of the appropriate level for tests and full reporting of outcomes
☒	☐	Estimates of effect sizes (e.g. Cohen's d, Pearson's r), indicating how they were calculated

Our web collection on statistics for biologists contains articles on many of the points above.

Software and code

Policy information about availability of computer code

Data collection	In order to obtain cell tracking data, MATLAB custom code was employed. The description on the cell tracking is explained in Method section.
Data analysis	In order to quantify the directionality of collective cell migration using polar histogram, ImageJ was employed to convert into binary images and extract cell existing area. Then Matlab was employed to calculate the angles as well as the simulation for the cellular behaviour. The detailed descriptions was shown in Method section and Supplementary documents.

For manuscripts utilizing custom algorithms or software that are central to the research but not yet described in published literature, software must be made available to editors and reviewers. We strongly encourage code deposition in a community repository (e.g. GitHub). See the Nature Research guidelines for submitting code & software for further information.

Data

Policy information about availability of data

All manuscripts must include a data availability statement. This statement should provide the following information, where applicable:

- Accession codes, unique identifiers, or web links for publicly available datasets
- A list of figures that have associated raw data
- A description of any restrictions on data availability

All data generated or analysed during this study are included in this published article (and in the supplementary information files).

Field-specific reporting

Please select the one below that is the best fit for your research. If you are not sure, read the appropriate sections before making your selection.

- [x] Life sciences
- [ ] Behavioural & social sciences
- [ ] Ecological, evolutionary & environmental sciences

For a reference copy of the document with all sections, see nature.com/documents/nr-reporting-summary-flat.pdf

Life sciences study design

All studies must disclose on these points even when the disclosure is negative.

Sample size	For each group that was compared statistically, the sample size was more than 5.<br>All experiments were conducted under environmental control by engineering technologies, and the variation is small enough for static analysis.
Data exclusions	No data was excluded
Replication	All attempts of replication were successful.
Randomization	The environment in the in vitro experiments were controlled by microfabrication technologies, which is described in the paper, to reduce the randomness.
Blinding	We are blinded to group allocations during data collection.

Reporting for specific materials, systems and methods

We require information from authors about some types of materials, experimental systems and methods used in many studies. Here, indicate whether each material, system or method listed is relevant to your study. If you are not sure if a list item applies to your research, read the appropriate section before selecting a response.

Materials & experimental systems

n/a	Involved in the study	
[ ]	[x]	Antibodies
[ ]	[x]	Eukaryotic cell lines
[x]	[ ]	Palaeontology and archaeology
[x]	[ ]	Animals and other organisms
[x]	[ ]	Human research participants
[x]	[ ]	Clinical data
[x]	[ ]	Dual use research of concern

Methods

n/a	Involved in the study	
[x]	[ ]	ChIP-seq
[x]	[ ]	Flow cytometry
[x]	[ ]	MRI-based neuroimaging

Antibodies

Antibodies used	A rabbit polyclonal anti-laminin antibody (L9393 MilliporeSigma), a mouse monoclonal Anti-Fibronectin antibody (F0916, MilliporeSigma), Recombinant Anti-E Cadherin antibody (ab40772, abcam)
Validation	All antibodies used in this paper were validated in IF and WB by manufactures.

Eukaryotic cell lines

Policy information about cell lines

Cell line source(s)	Normal Human Bronchial Epithelial cells: LONZA inc. (catalog #: CC-2541)<br>Madin-Darby Canine Kidney cells: RIKEN BioResource Research Center (product #: RCB0995)
Authentication	None of the cell lines used were authenticated.
Mycoplasma contamination	All cell lines were tested negative for mycoplasma by the suppliers.
Commonly misidentified lines<br>(See ICLAC register)	*Name any commonly misidentified cell lines used in the study and provide a rationale for their use.*
